# Structural brain disconnectivity mapping of post-stroke fatigue

**DOI:** 10.1016/j.nicl.2021.102635

**Published:** 2021-03-22

**Authors:** Kristine M. Ulrichsen, Knut K. Kolskår, Geneviève Richard, Dag Alnæs, Erlend S. Dørum, Anne-Marthe Sanders, Sveinung Tornås, Jennifer Monereo Sánchez, Andreas Engvig, Hege Ihle-Hansen, Michel Thiebaut de Schotten, Jan E. Nordvik, Lars T. Westlye

**Affiliations:** a NORMENT, Division of Mental Health and Addiction, Oslo University Hospital & Institute of Clinical Medicine, University of Oslo, Norway; b Department of Psychology, University of Oslo, Norway; c Sunnaas Rehabilitation Hospital HT, Nesodden, Norway; d Bjørknes College, Oslo, Norway; e Department of Neurology, Oslo University Hospital, Norway; f Brain Connectivity and Behaviour Laboratory, Sorbonne Universities, Paris, France; g Groupe d’Imagerie Neurofonctionnelle, Institut Des Maladies Neurodégénératives- UMR 5293, CNRS, CEA University of Bordeaux, Bordeaux, France; h CatoSenteret Rehabilitation Center, Son, Norway; i Faculty of Health, Medicine and Life Sciences, Maastricht University, Netherlands; j Department of Radiology and Nuclear Medicine, Maastricht University Medical Center, Netherlands; k Department of Nephrology, Oslo University Hospital, Ullevål, Norway; l KG Jebsen Centre for Neurodevelopmental Disorders, University of Oslo, Norway

**Keywords:** Stroke, Post-stroke fatigue, MRI, Brain mapping, Structural disconnectome, Lesion

## Abstract

Stroke patients commonly suffer from post stroke fatigue (PSF). Despite a general consensus that brain perturbations constitute a precipitating event in the multifactorial etiology of PSF, the specific predictive value of conventional lesion characteristics such as size and localization remains unclear. The current study represents a novel approach to assess the neural correlates of PSF in chronic stroke patients. While previous research has focused primarily on lesion location or size, with mixed or inconclusive results, we targeted the extended structural network implicated by the lesion, and evaluated the added explanatory value of a structural disconnectivity approach with regards to the brain correlates of PSF. To this end, we estimated individual structural brain disconnectome maps in 84 S survivors in the chronic phase (≥3 months post stroke) using information about lesion location and normative white matter pathways obtained from 170 healthy individuals. PSF was measured by the Fatigue Severity Scale (FSS). Voxel wise analyses using non-parametric permutation-based inference were conducted on disconnectome maps to estimate regional effects of disconnectivity. Associations between PSF and global disconnectivity and clinical lesion characteristics were tested by linear models, and we estimated Bayes factor to quantify the evidence for the null and alternative hypotheses, respectively. The results revealed no significant associations between PSF and disconnectome measures or lesion characteristics, with moderate evidence in favor of the null hypothesis. These results suggest that symptoms of post-stroke fatigue among chronic stroke patients are not simply explained by lesion characteristics or the extent and distribution of structural brain disconnectome, and are discussed in light of methodological considerations.

## Highlights

• We tested for associations between post stroke fatigue (PSF) and both lesion characteristics and brain structural disconnectome in 84 S patients.
• Results provided no evidence supporting a simple association between PSF severity and lesion characteristics or disconnectivity.
• PSF was strongly correlated with depression.
• Further studies including patients with more severe symptoms are needed to generalize the findings across a wider clinical spectrum.

## 1 Introduction

Between 25 and 85 percent of stroke survivors experience post stroke fatigue (PSF) (Cumming, Packer, Kramer, & English, 2016), described as an excessive and debilitative tiredness that can be unrelated to strain and not ameliorated by rest (UK Stroke Association, 2020, de Groot et al., 2003). Persistent PSF can be highly distressing, negatively impacting quality of life (de Bruijn et al., 2015, Naess et al., 2006) and preventing social participation and attendance to rehabilitation programs (Nadarajah & Goh, 2015). PSF is associated with both poor functional outcome and increased mortality (Glader, Stegmayr, & Asplund, 2002), and a recent *meta*-analysis revealed that the prevalence increases with time since stroke (Cumming et al., 2018). Early detection, prevention and treatment of fatigue might thus have positive effects on the overall outcome of stroke rehabilitation and quality of life. As such, identification of risk factors is important to facilitate detection and individual tailoring of rehabilitation programs.

PSF is considered a multifactorial condition with a complex etiology (Choi-Kwon & Kim, 2011). Among the most commonly reported risk factors are depression (Ponchel et al., 2015, Wu et al., 2014), reduced physical function (Lerdal et al., 2011, Aarnes et al., 2019), anxiety (Cumming et al., 2018, Wu et al., 2014), various medications (Chen & Marsh, 2018), pain and sleep disturbances (Naess, Lunde, Brogger, & Waje-Andreassen, 2012). While PSF is generally conceptualized as an independent condition, the clinical overlap with depression is substantial (Cumming et al., 2018), and the nature of the relationship between fatigue and depression has been debated. The use of advanced brain imaging to detect the brain correlates of the two clinical syndromes may facilitate our understanding of the phenomena through identification of both common and specific brain mechanisms (Høgestøl et al., 2019).

Despite a general consensus that the lesion and the associated brain perturbations following the stroke constitute causal factors for PSF, little is known about the predictive value of key lesion characteristics such as extent and neuroanatomical distribution. Fatigue is more prevalent following a minor stroke compared to a transient ischemic attack (TIA) (Naess et al., 2012, Winward et al., 2009), suggesting that the vascular lesion itself is of importance with regards to fatigue. Further, stroke survivors describe the fatigue experienced after stroke as qualitatively different than fatigue before stroke or normal tiredness (Thomas, Gamlin, De Simoni, Mullis, & Mant, 2019). Lastly, fatigue is a common sequela or symptom in several neurological conditions, i.e. traumatic brain injury, multiple sclerosis and postpoliomyelitis, jointly referred to as “central fatigue” (Chaudhuri and Behan, 2000, Chaudhuri and Behan, 2004).

Studies examining associations between lesion characteristics and fatigue in stroke survivors have generated mixed findings. In line with the hypothesis of fatigue caused by nervous system disruptions (Chaudhuri and Behan, 2000, Chaudhuri and Behan, 2004), basal ganglia infarcts have been identified as predictors of fatigue (Tang et al., 2010) and caudate infarcts were more frequent in patients with, than without, PSF (Tang et al., 2013). Further, infratentorial infarcts have been associated with increased risk of fatigue (Snaphaan, Van der Werf, & de Leeuw, 2011), as have right hemisphere lesions, brainstem and thalamic lesions (Mutai, Furukawa, Houri, Suzuki, & Hanihara, 2017). Subcortical white matter lesions have been associated with PSF 15 months post-stroke (Tang et al., 2014), but the generalizability of these findings is unclear (Snaphaan et al., 2011). In short, the relationship between fatigue and lesion location remains unresolved (De Doncker, Dantzer, Ormstad, & Kuppuswamy, 2018), and several studies find no significant associations between lesion characteristics and fatigue (Choi-Kwon et al., 2005, Ingles et al., 1999, Mead et al., 2011).

It is conceivable that clinical symptoms following a stroke are not mediated primarily by the localization of the lesion, but rather by the functional neuroanatomy of the extended brain networks that are affected by the lesion and degree of preserved network function (Bartolomeo and Thiebaut de Schotten, 2016, Nordin et al., 2016, Thiebaut de Schotten et al., 2020). Neuroimaging suggests that many psychiatric and neurologic symptoms are related to complex brain networks of anatomically distant but connected regions (Fox, 2018) that are vulnerable to injuries in a range of locations. Through processes like diaschisis (remote neurophysiological changes or dysfunctions of a distant region caused by a focal injury (Carrera and Tononi, 2014, von Monakow, 1914)), disconnection (Geschwind, 1974) and transneuronal degeneration (Cowan, 1970), stroke lesions may affect brain function and behavior in ways not readily predicted by the location or size of the damaged tissue. For example, functional network disturbances have been observed between remotely connected cortical areas in both the unaffected and affected hemisphere (Rehme & Grefkes, 2013), and abrupted connectivity may cause impairments that are functionally similar to tissue necrosis (Bonilha et al., 2014), like when a patient suffers severe Brocas aphasia without any damage to Brocas area (Fridriksson et al., 2007). Probing the extended brain network characteristics involved in a lesion and its associations with outcome may therefore provide theoretically and clinically relevant information of the functional neuroanatomy of specific symptoms post stroke and other brain disorders.

Recent large-scale collaborative neuroimaging efforts have resulted in remarkable advances in the characterization of the human brain “connectome” and “disconnectome” (Thiebaut de Schotten et al., 2020), providing highly valuable roadmaps for studies attempting to link symptoms, lesions and brain networks. Notably, normative samples enable indirect estimations of structural and functional disconnection, by which individual lesions from clinical structural imaging can be embedded onto a template of functional or structural connections derived from healthy subjects, and the lesions’ effect on the global connectome is estimated by tracking the connections passing through the lesion (Salvalaggio et al., 2020). A key advantage of atlas based tractography methods is that they do not require costly and specialized imaging sequences beyond those routinely collected in the clinic (Boes et al., 2015), thus offering a versatile tool for clinical-neuroanatomical predictions in studies on brain lesions (Salvalaggio et al., 2020). While functional and structural (dis)connectivity are intimately connected, there are evidence suggesting that a lesion’s impact on functional connectivity is primarily determined by how the lesion affects the structural connectome (Griffis et al., 2019), and indirect measures of structural disconnection have been found to perform significantly better than indirect measures of functional disconnection in predicting behavior (Salvalaggio et al., 2020).

By date, the relationship between PSF and stroke lesions has not been evaluated using a structural disconnectome approach, but a recent study on fatigue in multiple sclerosis (MS) revealed associations between structural network disconnection and subjective fatigue severity beyond what was explained by conventional MRI measures (Fuchs et al., 2019). With regards to the inconsistent findings on the relationship between stroke lesions and PSF, targeting structural network disconnections in addition to lesion characteristics may thus have the potential to advance our understanding on the relationship between brain perturbations and fatigue beyond what is revealed by traditional lesion-symptom mapping.

To evaluate the added explanatory value of a disconnectivity based approach with regards to the brain correlates of PSF, we quantified lesion disconnectivity indirectly using information about normative white matter pathways in the healthy population to estimate individual structural disconnection (disconnectome) maps in 84 S survivors in the chronic phase. The maps were created by a tractography-based procedure (Foulon et al., 2018) yielding voxel-wise probability of structural disconnection of white matter tracts (Salvalaggio et al., 2020).

Associations between disconnectome maps and PSF (assessed by the Fatigue Severity Scale (FSS)), were examined using permutation testing. Due to the substantial overlap and interaction between fatigue and depression and the possibility of common mechanisms across these conditions, all voxel-wise analyses were done with a) fatigue scores, b) depression scores (measured using the Pittsburg Health Questionnaire (PHQ-9) (Spitzer et al., 1999) fatigue and depression scores combined. The above described disconnectome based analyses were repeated on the binarized lesion maps, reflecting a traditional voxel-based lesion symptom mapping (VSLM) approach (Bates et al., 2003). In addition, we estimated the global disconnectivity for each patient, and tested for correlations with FSS, using Bayes factor to quantify evidence for the null hypothesis. Finally, in agreement with a more traditional, clinical approach, we applied linear models to test for associations between PSF and stroke related factors such as stroke location (right hemisphere, left hemisphere, brainstem, cerebellum, or both hemispheres), months since stroke, stroke severity (using National Institute of Health Stroke Scale (NIHSS; Lyden et al., 2009) score at discharge as a proxy for clinical severity) and etiology as defined by the stroke subtype classification system Trial of Org 10,172 in Acute Stroke Treatment (TOAST; Adams et al., 1993).

Due to a lack of previous studies applying a disconnectivity approach to PSF, we remained agnostic about the specific brain networks involved and performed a whole-brain analysis. Based on recent work demonstrating the benefits of targeting network projections of a lesion (Griffis et al., 2019, Thiebaut de Schotten et al., 2020), and the notion that many psychiatric and neurological conditions correspond more closely to brain networks than specific regions (Fox, 2018), we expected the disconnectivity based approach to demonstrate higher sensitivity to PSF than conventional lesion-related approaches.

## 2 Materials and methods

### 2.1 Study participants

Table 1 summarizes demographic and clinical information for the patient sample and the healthy control group.

**Table 1:** 

*Sample characteristics*	Patients (n = 84)		Control group (n = 155)		t (p)	BF**
*Demographic and clinical information*	Mean (SD)	Min-Max	Mean(SD)	Min-Max		
Age	65.8 (12.6)	24–87	64.7(12.3)	24–92	1.0 (0.279)	0.16
Males/females (count)	60/24		111/44			
Education in years	14.5 (3.4)	7–30	15.7 (3.3)	6–25	0.6 (0.513)	0.6
FSS	3.9 (1.5)	1–7	2.9 (1.3)	1–6	−3.9 (<0.001)	1911
PHQ-9	5.0(4.5)	0–21	3.2 (3.0)	0–15	−3.2 (0.001)	30
Montreal cognitive assessment (MoCA)	26.3(2.4)	19–30	27.4 (1.7)	22–30	3.0 (0.002)	171

*Stroke related patient information*						
NIHSS at hospital discharge	1.1 (1.2)	0–6				
TOAST classification for ischemic stroke*	Large artery artherosclerosis (26)					
	Small vessel occlusion (26)					
	Cardioembolism (13)					
	Other determined etiology (6)					
	Undetermined etiology (13)					
Lesion location	Brainstem/cerebellum (17)					
	Left Hemisphere (26)					
	Right Hemisphere (34)					
	Both Hemispheres (6)					
Months since stroke	22.0 (11.9)	3–45				

*all but one patient suffered ischemic stroke

**BF = Bayes factor.

Both frequentist and Bayesian statistics are reported, in line with current pragmatic recommendations (Keysers et al., 2020).

#### 2.1.1 Healthy control group

Healthy individuals > 18 years were recruited through newspaper ads, word-of-mouth and social media (Dørum et al., 2020, Richard et al., 2018). Exclusion criteria for healthy controls included a history of stroke, neurological or psychiatric disease, medications with significant effects on central nervous system function and MR contraindications.

Healthy controls and stroke patients were matched on age and sex, using *MatchIt* in R (Stuart, King, Imai, & Ho, 2011) and the default method *nearest*. Applying a ratio of 2:1 (two controls selected for each patient), healthy participants were collected from a pool of 341 controls (age 24–92), resulting in an age- and sex matched control group of 155 individuals (mean age = 64.7, SD = 12.3, 44 females).

#### 2.1.2 Patients

We recruited 84 S patients from the Geriatric Department, Diakonhjemmet Hospital, the Stroke Unit, Oslo University Hospital and Bærum Hospital. A subsample of the patients (n = 66) participated in a longitudinal intervention study examining the effects of cognitive training and tDCS on cognitive function (see Kolskaar et al. (2020) for details). All data reported in the current study were collected prior to the intervention. Criteria were ischemic or hemorrhagic stroke in a chronic phase defined as ≥ 3 months since admission, age above 18 years, no MRI contraindications and no other known, severe neurological conditions prior to the stroke. While aphasia was not a formal exclusion criterion and was not assessed explicitly, no patients reported or revealed severe speech or language impairments. All participants provided informed consent prior to enrollment. The study was approved by the Regional Committee for Medical and Health Research Ethics, South-East Norway.

## 3 Clinical measures

Stroke subtype was classified by the Trial of Org 10,172 in Acute Stroke Treatment (TOAST; (Adams et al., 1993), and clinical assessment of stroke severity was indexed by the National Institute of Health Stroke Scale (NIHSS) at hospital discharge.

Subjective fatigue was measured by the Fatigue Severity Scale (FSS) (Krupp, LaRocca, Muir-Nash, & Steinberg, 1989), which is a self-report scale consisting of 9 statements about impact of fatigue on daily life. Degree of agreement is indicated on a seven-point Likert scale (lowest possible total score 7, highest score 63). FSS is one of the most frequently used instruments for measuring fatigue in neurological conditions (Cumming et al., 2016) and has demonstrated reasonable psychometric qualities (Whitehead, 2009). A commonly adapted threshold for clinical fatigue is a mean score of >= 4 (total score >= 36) (Krupp et al., 1989, Nadarajah and Goh, 2015, Schepers et al., 2006), where a higher score is suggested to indicate a moderate to high impact of fatigue (Schepers et al., 2006).

Depressive symptoms were measured by the depression module of the PHQ-9, in which occurrence of depressive symptoms corresponding to the DSM-IV criteria is rated on a 9-item Likert scale. Scores range from 0 (not at all) to 3 (nearly every day), yielding a minimum score of zero and a maximum score of 27. A cutoff score of ≥ 10 has demonstrated acceptable sensitivity and specificity for depression (Kroenke et al., 2001). Cognition was measured by Montreal Cognitive Assessment (MoCA) (Nasreddine et al., 2005), and sleep quality was assessed by the Pittsburgh Sleep Quality Index (PSQI) (Buysse et al., 1989).

## 4 MRI acquisition

Patients were scanned at Oslo University Hospital on a 3 T GE 750 Discovery MRI scanner with a 32-channel head coil. We collected structural (T1w, FLAIR), functional (resting-state and task-based fMRI) and diffusion MRI data. For lesion demarcation used in the present analysis T1-weighted images were collected using a 3D IR-prepared FSPGR (BRAVO) sequence (TR: 8.16 ms; TE: 3.18 ms; TI: 450 ms; FA: 12°; voxel size: 1 × 1 × 1 mm; slices: 188; FOV: 256 × 256, 188 sagittal slices), and T2-FLAIR with the following parameters: TR: 8000 ms; TE: 127 ms, TI: 2240; voxel size: 1 × 1 × 1 mm).

## 5 Lesion demarcation

**Fig. 1:**
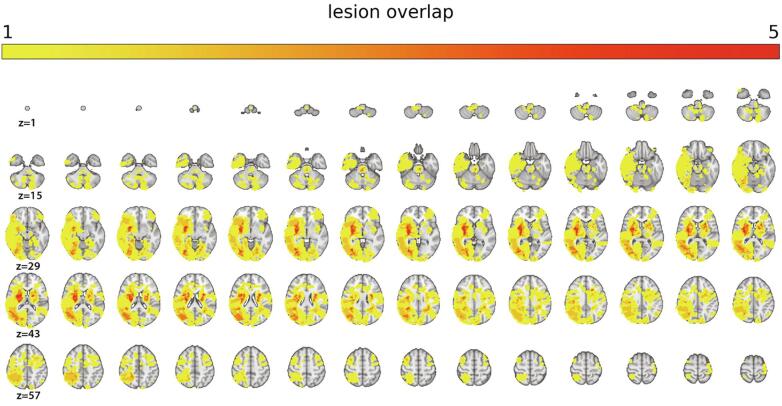
Heatmap displaying lesion overlap across stroke patients by 70 slices (2 mm thickness) from z(voxel) = 1 to z = 70. Maximal overlap was 8, but for illustration purposes, the color scale saturates at 5.

Lesions were demarcated in native space, using the Clusterize toolbox (de Haan et al., 2015) with SPM8 running under Matlab R2013b (The Mathworks, Inc., Natick, MA). Lesions were traced by trained personnel (a physician and a radiographer), based on hyperintensities and visible damage on FLAIR images, and guided by independent neuroradiological descriptions of dMRI/FLAIR images (see Dørum et al. (2020) for details). The binarized lesion masks were registered to MNI space using nearest neighbor interpolation, using the transformation parameters obtained using the T1w data. To register the FLAIR images to the T1 images, we applied a linear transformation with 6 degrees of freedom. T1 images were registered to MNI152 space by linear affine transformation with 12 degrees of freedom. Fig. 1 displays a probabilistic map of lesion overlap across patients.

### 5.0.1 Disconnectome maps

To calculate the disconnectome maps we used an automated tractography-based procedure (Foulon et al., 2018) implemented in the *BCBtoolkit disconnectome maps* (Brain Connectivity Behaviour Toolkit (BCBtoolkit)). Briefly, a training set based on full-brain tractography data obtained from a normative group of 170 individuals from the Human Connectome Project 7 T data (HCP 7 T) was used to track fibers passing through each lesion. Using affine and diffeomorphic deformations (Avants et al., 2011, Klein et al., 2009), each patient́s MNI 152 space lesions were registered to each controĺs native space, and used as seed for the tractography in Trackvis (Wang et al., 2007). Subsequently, the tractography was transformed to visitation maps, binarized and registered to MNI152 space, before a percentage overlap map was produced by summarizing each point in the normalized healthy subject visitation maps. The resulting disconnectome maps indicate a voxel-wise probability of lesion-related disconnection ranging from 0 to 100%. We computed two simple summary measures of disconnection severity, defined for each patient as a) mean voxel intensity across the individual disconnectome map and b) number of voxels within the individual disconnectome map with intensity > 0.5 (reflecting 50% probability of disconnection).

#### 5.1 Statistical analysis

Voxel wise analyses on disconnectome maps and binarized lesions (VLSM) were done by non-parametric permutation-based inference as implemented in the FSL randomise tool (Winkler et al., 2014). The statistical tests subsequently described were repeated in separate models for disconnectome maps and binarized lesions alike. Within the framework of the general linear model (GLM), linear effects of fatigue and depression (indicated by total score on FSS and PHQ, respectively) were tested in separate models, covarying for age and sex. To comply with a more common clinical definition of PSF, we re-ran the model on dichotomized fatigue variables defined as either a) a mean FSS score of ≥ 4, consistent with the common cut off value, or b) the upper tertile of FSS total score (contrasted with the lowest tertile), reflecting the possibility that more extreme scores demonstrate increased sensitivity to fatigue related brain correlates. We estimated models controlling for depression in two different ways, first by excluding patients scoring above clinical threshold on PHQ (remaining n = 74), and second, by including z-normalized summary scores from both FSS and PHQ in the same model. One additional model tested the effect of fatigue and depression combined, applying the total of the z-normalized sum scores (zPHQ + zFSS) as predictor. For each contrast, 5000 permutations were performed. Results were thresholded by threshold free cluster enhancement (TFCE, Smith and Nichols (2009)) and considered significant at p < 0.05, two tailed, corrected for multiple comparisons using permutation testing. One patient suffered a very large stroke and constituted an outlier in terms of number of affected voxels (~8 SDs above the mean). Main analyses were therefore repeated with this patient excluded.

Subsequent statistical analyses were performed using R version 3.4.0 (R Core Team, 2017). In a follow-up analysis aiming to increase sensitivity to clinical measures and evaluate the relationship between global disconnectivity and fatigue, we computed two disconnection severity measures, defined for each patient as a) mean voxel intensity across the individual disconnectome map and b) number of voxels within the individual disconnectome map with intensity > 0.5 (reflecting 50% probability), and correlated these with FSS and PHQ-9. To quantify the evidence in favor of the null and alternative hypothesis, we applied Bayes factor hypothesis testing, in line with current recommendations (Keysers et al., 2020). We applied the BayesFactor package (Morey et al., 2015) with default priors. For transparency, key analyses were run with different priors.

To test for associations with clinical, stroke-related characteristics (TOAST classification, months since stroke, lesion volume and lesion location), we applied linear models with FSS score as dependent variable, controlling for age and depressive symptoms. Lesion location was clustered by four categories – right or left hemisphere, both hemispheres or brainstem/cerebellum. Stroke variables were added subsequently, allowing for model comparison by Bayes factor for each added variable. We applied the lmBF function from the BayesFactor package to compute Bayes factors. As an additional test of the added predictive value of global disconnectivity measures compared to clinical stroke characteristics, we also estimated the models with mean voxel intensity across the individual disconnectome map and number of voxels within the individual disconnectome map with intensity > 0.5.

## 6 Results

### 6.0.1 Fatigue and depression in the stroke sample compared to healthy controls

**Fig. 2:**
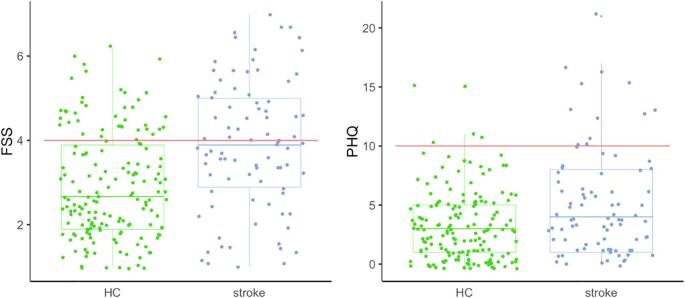
Distributions and group differences in FSS and PHQ for healthy controls (HC) and stroke patients. Red line denotes cut off value for clinically significant symptom load. (For interpretation of the references to color in this figure legend, the reader is referred to the web version of this article.)

Fig. 2 shows the distributions of FSS and PHQ score in each group. 48 percent of the stroke patients reported clinically significant fatigue (mean FSS > 4), compared to 23 percent of the control participants. Severe fatigue (mean FSS > 6) was reported by 9 percent of the patients and 1 percent of the healthy controls. A two-tailed, two sample *t*-test (ttestBF in BayesFactor, with default Cauchy prior) provided compelling evidence (Bayes Factor: BF) > 150) for higher total FSS scores in the patient group (mean = 35) compared to healthy controls (mean = 26, median posterior *δ* = −8.5, 95% credible interval (CI) = [−11–−5 ]), relative to the null hypothesis. Stroke patients (mean = 5.0) also reported higher levels of depression symptoms on the PHQ than controls (mean = 3.2). 18 percent of the patients scored 10 or higher, indicating clinical depression, compared to 2.4 percent of the controls. The corresponding Bayes factor provided strong evidence for a group difference in PHQ sum score (BF = 20, 95% CI = [−2.5–−0.5]).

### 6.0.2 Fatigue associations in patient sample

Among patients, Bayes factor estimation for linear correlations provided strong evidence (BF > 150) for a positive association between FSS and PHQ (median posterior *δ* = 0.71, 95% CI = [0.59–0.80]), suggesting more depressive symptoms with increasing fatigue. The substantial association persisted when re-estimating the correlation after excluding the fatigue-item (“feeling tired or having little energy”) from the PHQ scale (median posterior *δ* = 0.61, 95% CI = [0.46–0.73]). There was only anecdotal support for an association between FSS and age (BF = 1.20, median posterior *δ* = -0.18, 95% CI = [−0.38–0.02]). Mean global PSQI score was 6.8 (SD = 3.6), with 51 patients (60%) scoring > 5, indicating poor sleep quality in a majority of patients. Global PSQI correlated moderately with FSS score (BF > 150, median posterior *δ* = 0.49, 95% CI = [0.31–0.64]), and strongly with PHQ score (BF > 150, median posterior *δ* = 0.61, 95% CI = [0.47–0.73]).

## 7 Permutation based analyses on disconnectome maps and lesions

**Fig. 3:**
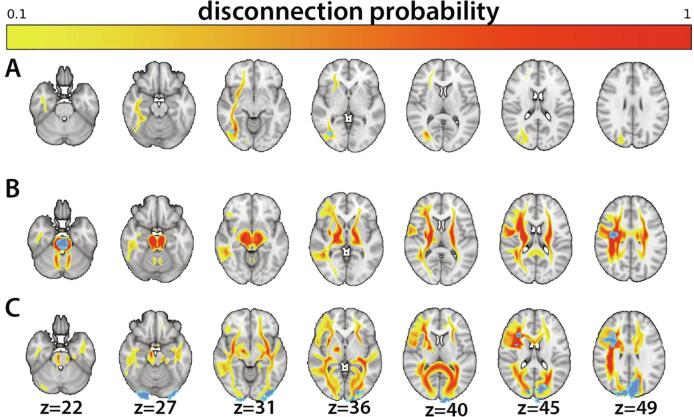
Individual lesions (blue) and associated disconnectome maps (yellow–red). Probability for disconnection ranges from 10 (yellow) to 100 (red). Patient **A**: right cerebral white matter lesion, Patient **B:** brain stem lesion, Patient **C:** left and right cerebral cortex and white matter lesions. (For interpretation of the references to color in this figure legend, the reader is referred to the web version of this article.)

Fig. 3 shows a selection of stroke lesions and the associated disconnectome maps, for illustrative purposes.

Permutation testing revealed no significant associations between the disconnectome maps and the clinical measures (FSS, PHQ-9, FSS/PHQ combined), or of fatigue status defined by either a) mean FSS score of ≥ 4, or b) by the lowest vs highest FSS tertile. Controlling for depression by a) excluding patients with depression from the analysis or b) including PHQ scores in the model revealed similar results.

Similarly, permutation tests on binarized lesion maps (voxel-based lesion symptom mapping) revealed no significant associations with the clinical measures (fatigue, depression or fatigue/depression combined, or on group defined by fatigue status (mean FSS score ≥ 4). Due to the considerable reduction in sample size when including only the lowest and highest FSS tertile (n = 56), we did not repeat this analysis on the binarized lesion maps. For transparency, the distributions of the uncorrected t-values across the brain from the models testing for associations with either disconnectome or lesion maps are depicted in Supplementary Fig. 1, and the corresponding number of uncorrected voxels with p < 0.05 are reported in Supplementary Table 1.

### 7.1 Associations between global summary measures of disconnectivity and clinical measures

Both measures of global disconnectivity (mean value in disconnectome map and number of voxels with disconnection probability > 50%) were strongly correlated with lesion size (posterior mean = 0.74, BF > 150 and median posterior *δ* = 0.68, BF > 150, respectively). Global disconnectivity (mean value in disconnectome map) was not correlated with FSS (median posterior *δ* = 0.03) or PHQ (median posterior *δ* = 0.03). Bayesian correlations (using default priors) provided moderate evidence (BF = 0.26) for these null effects, relative to H1 (positive associations between disconnectivity measures and FSS/PHQ). This indication of a null effect was mirrored in correlations between the alternative measure of disconnectivity (number of voxels with disconnection probability > 50%) and FSS (median posterior *δ* = 0.05, BF = 0.29), and PHQ (median posterior *δ* = 0.02, BF = 0.26). For transparency, Bayes factors of the main correlations estimated on different priors are reported in Supplementary Table 2, while Supplementary Table 3 reports the correlations after removing the most extreme outlier in terms of lesion size.

### 7.2 Associations between clinical stroke-related characteristics and FSS

Linear models (lmBF) corrected for age and depressive symptoms did not provide evidence for associations between FSS scores and lesion location (brainstem/cerebellum, left or right hemisphere or both hemispheres), lesion volume, months since stroke or TOAST stroke classification (see Supplementary Table 4 for model comparisons and associated Bayes factors). No stroke related variable, including global disconnectivity, demonstrated Bayes factors > 1, indicating low predictive value for all. Specifically, all extended models with stroke lesion variables displayed Bayes factors below 0.33 when compared to the simpler null model, suggesting moderate evidence of no effect of stroke lesion characteristics.

## 8 Discussion

Fatigue following stroke is common and represents a significant clinical burden. Stroke sequelae reflect both cell death at the site of the lesion, as well as structural and functional alterations in extended brain networks. Brain network dysfunction, directly or indirectly related to the stroke lesion, is a putative mechanism underlying PSF pathophysiology. Previous studies have primarily assessed lesion characteristics such as volume or location, and the added explanatory value of probing the extended brain network connections with the lesion has been unclear. To this end, we calculated structural disconnectome maps for 84 patients in the chronic phase and used permutation testing to evaluate the association between PSF symptoms and regional network disconnection.

Permutation testing revealed no significant associations between symptoms of fatigue and disconnectome maps, or between fatigue and binarized lesion maps (VLSM). We found no support for our hypothesis that a disconnectivity approach by disconnectome maps would add predictive value of fatigue beyond conventional lesion analyses. In line with this, Bayes factor estimations on correlations between disconnectivity summary measures and FSS score provided moderate evidence for the null hypothesis (no association) relative to the alternative hypothesis (association between fatigue and disconnectivity). However, results are not decisive, and alternative explanations of the absent effects must be considered.

The lack of added predictive value from the disconnectivity measures when compared to more traditional lesion characteristics is in general agreement with recent studies (Hope et al., 2018, Salvalaggio et al., 2020) reporting similar predictive value for models including (dis)connectivity measures compared to models with lesion information only. The lack of robust associations between disconnectome maps and the clinical measures has several likely explanations. It has been suggested that the information provided in the disconnectome maps is largely embedded in the binarized lesion masks (Hope et al., 2018, Salvalaggio et al., 2020), implying that the two representations of lesion related pathology convey overlapping variance. Indeed, the correlation between lesion volume and global disconnectivity, operationalized as the average voxel value across the disconnectome map or the number of voxels with probability of disconnection > 50%, was relatively strong, intuitively supporting that larger lesions project to a larger proportion of the brain.

Alternatively, it may be that disconnectome maps and lesion masks convey similar information primarily when the sample is large and lesion diversity sufficiently high to capture the variance embedded in the disconnectome maps (Griffis et al., 2019). This could imply that in many real-life situations where large samples are not always realistic/feasible, such as in clinical stroke populations, disconnectome maps may provide relevant, complementary and unique information. In agreement with this, a recent study revealed that structural disconnectome maps explained a larger proportion of the variance in core functional connectivity disruptions than did focal lesions, and displayed significant correlations with behavior (Griffis et al., 2019), thus facilitating the understanding of individual differences in outcome. Moreover, higher accuracy in cognitive and mobility prediction for models including disconnection metrics than models based on lesion volume has been reported (Kuceyeski et al., 2016).

A key assumption underlying our analyses is that the indirectly calculated disconnectome maps provide a realistic estimate of structural network disconnection and that these disconnections have functional effects. As depicted in Fig. 3, the degree of estimated tract disconnection can be extensive, even for smaller lesions. While such lesion to brain network mapping supports the notion that lesions in hub-like regions project to and implicates an extended set of brain regions and networks (Colizza et al., 2006, van den Heuvel and Sporns, 2011), the tractography process used to build the normative training set has several inherent limitations and errors can be introduced in any stage of the tracking process (Schilling et al., 2019). Noise and artefacts in the image acquisition, difficulties establishing fiber orientation (Jeurissen et al., 2019) and choices regarding the tracking algorithm and parameters such as stop criteria and curvature threshold (Knösche et al., 2015, Schilling et al., 2019), are among the commonly recognized pitfalls. Consequently, the reconstructed pathways based on diffusion tractography may not necessarily reflect true structural connections, and to which degree disconnectome maps reflect biological disconnections is still debated (Salvalaggio et al., 2020), warranting caution when interpreting tractography results without supporting converging evidence (Jeurissen et al., 2019). These limitations are not specific for the currently employed algorithm, and further work is needed to overcome general limitation of biological accuracy and validity of diffusion based tractography. On a related note, different approaches for disconnectome mapping may reveal different associations with clinical measures, which should be pursued in future validation studies.

The added value of disconnectome maps in brain-behavior mapping also depends on the reliability, validity and functional neuroanatomy of the included clinical and behavioral measures. For example, primary motor dysfunctions, which may require simpler operationalizations and measurements than more complex cognitive symptoms, are more strongly associated with focal damage, while other behaviors, like verbal associative memory, may be more strongly predicted by extended network function (Griffis et al., 2019, Siegel et al., 2016). The lack of significant associations between brain characteristics and behavioral measures in the current study may therefore be partly related to the properties and measurement of PSF. Although fatigue is painfully tangible for the individual patient, it is unspecific and difficult to operationalize, and the lack of “gold standard” measures of subjective fatigue has been characterized as one of the major obstacles to PSF research (Nadarajah & Goh, 2015). In the current study, we applied the FSS as a general measure of fatigue interference and severity. As FSS is the most widely used fatigue measure in stroke research (Cumming et al., 2016), reporting FSS scores facilitates communication and synthesizing of results across studies. Still, FSS constitutes a rather coarse measure of a complex phenomenon, and does not provide information on other relevant aspects of PSF such as diurnal fluctuations and fatigue subtypes. It is conceivable that more finely grained measures of i.e. fatigue subtypes could reveal associations not detected by the FSS.

Mimicking the results from the disconnectome approach, linear regressions testing for associations between FSS and lesion characteristics (volume and location) revealed no significant associations. This is in agreement with several previous studies (Choi-Kwon et al., 2005, Ingles et al., 1999, Mead et al., 2011). Still, the literature is inconclusive, and some suggest significant associations between PSF and lesion characteristics (Snaphaan et al., 2011, Tang et al., 2014, Tang et al., 2010). The inconsistency between studies may be attributable to differences in how lesion site is defined and reported, as well as time since stroke and clinical characteristics and severity of the sample. Studies that do not report significant associations between fatigue and lesion characteristics tend to define lesion location broadly (Wu et al., 2015), such as posterior/anterior circulation or left/right hemisphere, while studies reporting significant associations often apply a more detailed account of lesion site (e.g. which specific structures are affected) and are conducted within the first few months after stroke. The temporal aspect may be of particular importance, considering that the character of stroke sequelae and associated brain correlates change over time through processes of recovery and compensation (Fornito et al., 2015, Fox, 2018). In the present study, fatigue was measured on average 22 months post stroke. The absence of identified stroke lesion effects may thus suggest that lesion characteristics play a less critical role in the chronic phase (Aarnes et al., 2020).

In addition to the general limitations related to the interpretation of imaging-based measures of brain connectivity listed above, the results should be interpreted in light of the following caveats. First, the recruited patients suffered mild strokes and were drawn from a highly functioning part of the stroke population, as the extent and type of assessments prevented the more disabled patients from participating (e.g. severe aphasia, paralysis, severe neglect). This limits generalizability of results, and we cannot exclude the possibility that including more severely fatigued patients would reveal associations not detected in the current study. However, even in this sample of fairly high functioning stroke patients, levels of fatigue were significantly higher than in the healthy control group, and comparable to fatigue levels reported in other samples of chronic stroke patients (Choi-Kwon et al., 2005, Valko et al., 2008). Moreover, fatigue correlated highly with depression and moderately with sleep quality, in line with previous reports (Choi-Kwon et al., 2005, Park et al., 2009, van de Port et al., 2007), intuitively indicating that FSS scores reflect a relevant clinical phenotype.

Second, related to the complex and multifactorial nature of PSF, the design of the current study does not allow for an extensive account of all factors potentially involved in fatigue etiology. Unmeasured factors like pain (Naess et al., 2012, Tang et al., 2014), pre-stroke fatigue (Choi-Kwon et al., 2005, Duncan et al., 2014), social support (Michael, Allen, & Macko, 2006) and specific cognitive impairments like memory problems and reduced processing speed (Pihlaja et al., 2014, Ulrichsen et al., 2020) have been associated with PSF, as have the use of various medications (Chen & Marsh, 2018) (see e.g. Aarnes et al. (2020) for an updated review on PSF related factors). While the aim of this paper was to evaluate the added explanatory value of a structural disconnectome approach with regards to subjective fatigue, rather than providing an extensive mapping of associated factors and comorbidities, the number of potential confounders constitutes a principal constraint to the understanding of PSF etiology in our sample.

Third, VSLM analyses are inherently contingent on and restricted by the variability of the patients’ lesion locations, as a lesion site cannot be identified as important for a symptom if it is not represented in the sample. With regards to the current sample, the lack of whole brain representation limits the spatial scope of the analyses, where i.e. right hemispheric strokes were more densely sampled than left hemispheric strokes, and prefrontal cortex was marginally affected. This lack of full or random sampling of the brain represents a common caveat in stroke research, because stroke lesions are not randomly or evenly dispersed throughout the brain, but are dependent on vascular organization and architecture and tend to occur in proximity to major arteries (Rorden et al., 2007, Zhao et al., 2020). In line with this, degree of voxel-wise lesion overlap between patients in the current sample was low, and although a sample size of 84 is comparable with common practice in MRI studies targeting stroke (Nickel and Thomalla, 2017, Nott et al., 2019, Sihvonen et al., 2017), further studies with even larger samples are needed.

Low power due to small sample sizes is common in neuroscience (Button et al., 2013), and might be especially pressing in stroke imaging research where inter-patient variability in lesions and symptoms is high, and large datasets are logistically and financially demanding to collect (Liew et al., 2020, Price et al., 2017). With reference to this fundamental constraint, the best hope for future stroke neuroimaging studies may lie in large-scale data-sharing initiatives such as the ENIGMA Stroke Recovery database (Liew et al., 2020), where pooled and synthesized data from individual studies facilitates conduction of well powered studies on large and diverse samples.

However, in smaller samples with low lesion overlap, targeting disconnectivity through disconnectome maps may be particularly relevant, because such measures reveal common disruptions across spatially dispersed lesions (Griffis et al., 2019), resulting in a higher degree of disconnectome overlap compared to lesion overlap.

In conclusion, the current study represents a novel approach to assess the neural correlates of PSF in chronic stroke patients. By indirectly estimating structural network disconnections caused by the stroke lesions, we arrived at individual disconnectome maps capturing distal effects of focal damage. The results did not provide evidence that a structural disconnectome based approach demonstrates higher sensitivity to PSF than a VLSM approach. Nor did the results support the notion that lesions to particular regions or disconnections to specific networks contribute to PSF in the chronic phase. Importantly, our approach does not allow for claims about functional connectivity, and future studies should investigate whether the findings replicate with functional disconnectivity measures. Finally, methodological considerations regarding statistical power, lesion coverage- and overlap warrants caution when interpreting results.

## CRediT authorship contribution statement

**Kristine M. Ulrichsen:** Conceptualization, Formal analysis, Writing - original draft. **Knut K. Kolskår:** Data curation, Writing - review & editing. **Geneviève Richard:** Data curation, Writing - review & editing. **Dag Alnæs:** Visualization, Writing - review & editing. **Erlend S. Dørum:** Data curation, Writing - review & editing. **Anne-Marthe Sanders:** Data curation, Writing - review & editing. **Sveinung Tornås:** Supervision, Writing - review & editing. **Jennifer Monereo Sánchez:** Data curation, Writing - review & editing. **Andreas Engvig:** Writing - review & editing. **Hege Ihle-Hansen:** Writing - review & editing. **Michel Thiebaut de Schotten:** Methodology, Software, Writing - review & editing. **Jan E. Nordvik:** Project administration, Funding acquisition, Writing - review & editing. **Lars T. Westlye:** Conceptualization, Funding acquisition, Project administration, Supervision, Formal analysis, Writing - review & editing.

## Declaration of Competing Interest

The authors declare that they have no known competing financial interests or personal relationships that could have appeared to influence the work reported in this paper.
